# Wheat Fermentation With *Enterococcus mundtii* QAUSD01 and *Wickerhamomyces anomalus* QAUWA03 Consortia Induces Concurrent Gliadin and Phytic Acid Degradation and Inhibits Gliadin Toxicity in Caco-2 Monolayers

**DOI:** 10.3389/fmicb.2018.03312

**Published:** 2019-02-12

**Authors:** Hafiz Arbab Sakandar, Stan Kubow, Behnam Azadi, Rani Faryal, Barkat Ali, Shakira Ghazanfar, Umar Masood Quraishi, Muhammad Imran

**Affiliations:** ^1^School of Human Nutrition, Faculty of Agricultural and Environmental Sciences, McGill University, Sainte-Anne-de-Bellevue, QC, Canada; ^2^Department of Microbiology, Faculty of Biological Sciences, Quaid-i-Azam University, Islamabad, Pakistan; ^3^Food Sciences Research Institute, National Agricultural Research Centre, Islamabad, Pakistan; ^4^NIGAB, National Agricultural Research Centre, Islamabad, Pakistan; ^5^Department of Plant Sciences, Faculty of Biological Sciences, Quaid-i-Azam University, Islamabad, Pakistan

**Keywords:** yeast, bacteria, fermentation, Caco-2, celiac

## Abstract

Foods containing high amounts of either phytic acid or gliadin can pose a risk for development of iron deficiency and celiac disease, respectively. The present study was conducted to evaluate the effects of preselected gliadin degrading strains, *Enterococcus mundtii* QAUSD01 and *Wickerhamomyces anomalus* QAUWA03, on phytic acid and gliadin degradation in six wheat cultivars (*Lasani 2008*, *Seher 2006*, *Chakwal 97*, *Shafaq 2006*, *Bars 2009*, *Barani 83*). Tight junction proteins, *trans*-epithelial resistance (TER) and ruffle formation in Caco-2 cells were evaluated relative to *Saccharomyces cerevisiae*–mediated fermented and unfermented controls. Phytic acid degradation was demonstrated in all six cultivars fermented with *E. mundtii* QAUSD01 and *W. anomalus* QAUWA03 consortia. Among the six fermented cultivars, *Shafaq 2006* showed relatively higher degradation of gliadin. In comparison to the other tested wheat varieties, fermentation of *Lasani 2006* was associated with minimal toxic effects on Caco-2 cells in terms of ruffle formation, tight junction proteins and TER, which can be attributed to extensive degradation of toxic gliadin fragments.

## Introduction

Celiac disease (CD) results from a combination of gluten exposure and genetic factors (HLA; Human Leucocyte Antigen) and this disorder appears to be exacerbating with increasing numbers of sufferers (Green et al., 2015; Dowd and Jung, 2017). It is more prevalent in Europe and North America than in other regions such as Indonesia, South Korea, Philippines, and many smaller Pacific islands where it is rare, which is likely due to low wheat consumption together with a low frequency of HLA-DQB1^∗^02 (Jacobsson et al., 2017). In South-East Asia, HLA-DQB1^∗^02 has been noted to be present in more than 5% of the population but CD is rare, as the staple diet is based on rice. In contrast, prevalence rates of CD in Pakistan are similar to those observed in Europe, which is probably due to high dependency on gluten-based diets in Pakistan where wheat is a food staple. To date, the only treatment for CD patients is a lifelong gluten-free diet (Gujral et al., 2012). Several food technological techniques have been attempted to lower gluten levels in wheat products including sourdough fermentation (Moore and Gainer, 2014). Sourdough is a leavening agent that is traditionally obtained via a back-slopping procedure (Gänzle et al., 1998; Nionelli and Rizzello, 2016). Nowadays, novel biotechnological procedures have been adopted industrially to shorten the sourdough fermentation processing time such as the use of specifically selective lactic acid bacteria involving type II sourdough fermentation (Corsetti et al., 1998; Rinaldi et al., 2015). The use of lactic acid bacteria in sourdough fermentation improves the structure of bread, enhances the nutritional and organoleptic qualities of the ingredients, increases bioavailability of essential minerals and bioactive compounds and can also improve the glycemic response (Minervini et al., 2015; Ventimiglia et al., 2015). Among the various factors that affect the fermentation process, the most important are the nature of the microbes, type of flour, environment, modality of sourdough proliferation and fermentation parameters (Minervini et al., 2016). In particular, the appropriate selection of starter culture is important to enhance the functional and nutritional properties of the flour. The usage of a variety of industrial starter cultures has been restricted due to their limited capability to ferment various flour matrices (Coda et al., 2010).

In addition to the CD-related issues of gliadin in wheat, the presence of the anti-nutritional phytic acid factor in wheat-based cereals is also problematic (Kumar, 2016; Ahmad Mir and Ahmad Shah, 2018; Mehfooz et al., 2018). Excessive intake unleavened bread consisting of lesser refined wheat flour containing a high phytic acid content has been consistently related to an increased risk of various trace mineral deficiencies (Sarwar Gilani et al., 2012). In that regard, intake of wheat flour with a high phytic acid content is associated with chelation of iron, zinc and copper that leads to significantly decreased bioavailability (Hwalla et al., 2017). Iron deficiency is one of the prevailing nutrient deficiencies worldwide with reports of 800 million people suffering from this condition, particularly pregnant women and infants (McLean et al., 2009). High intake of phytic acid in cereal-based diets is one of the major risk factors for iron deficiency in developing countries (Qamar et al., 2015), which are more vulnerable to iron deficiency. For example, approximately 50% of non-pregnant women in Pakistan have been indicated to be iron deficient (Ullah et al., 2016). On the other hand, although whole grain cereals contain higher phytate content as compared to refined flours, whole grain products contain a significantly higher content of phytonutrients with health promoting benefits as compared refined cereal flours (Parmar et al., 2017).

Pakistan is well known for its vast production and consumption of cereal grains (Zuberi et al., 2016). Wheat production in Pakistan was about 26 million tons in 2016 and annual cereal production is increasing rapidly as an increase of 3% in production was recorded in 2015 (Food and Agriculture Organization of the United Nations [FAO], 2016). Pakistan is leading country for wheat consumption with a consumption rate of 24 million tons per year (USDA/FAS, 2016). Production of baked goods in Pakistan is increasing due to the improvement in national economic environment (Anjum et al., 2005). The most common food staple of Pakistan is wheat-based bread (Khan, 2014; Briones Alonso and Swinnen, 2016). Baker’s yeast is used for making bread at artisanal and industrial levels in Pakistan with seldom production of baked goods involving sourdough fermentation.

Since sourdough fermentation could be a feasible commercial approach to lower gluten and phytic acid content of baked wheat goods, the present study was performed to isolate and characterize the capability of lactic acid bacteria and yeast to concurrently degrade both sodium phytate and gluten. In addition, the potential for sourdough fermentation to reduce toxic effects of gliadin on human intestinal epithelial Caco-2 monolayers was assessed.

## Materials and Methods

### Microorganism and Cultural Conditions

In an earlier study (Sakandar et al., 2018), 19 microbial strains were isolated and screened for those that could maximally degrade gliadin. The study identified two gliadin-degrading yeast strains (*W. anomalus* QAUWA02 (KU949595.1) and *W. anomalus* QAUWA04 (KU949597.1) as well as several gliadin-degrading bacterial strains (*E. faecalis* QAUSD02 (KY785322.1), *E. faecalis* QAUSD04 (KY785324.1), *E. faecalis* QAUSD05 (KY785325.1), *E. faecalis* QAUSD06 (KY785326.1), *E. megaterium* QAUSD03 (KY785323.1) and *Bacillus cereus* QAUSD07 (KY785327.1) and *E. mundtii* QAUSD01 (KY785321.1). The bacterial strains were cultivated in MRTS broth at 37°C and the yeast strains had been cultivated in Sabouraud Dextrose Broth (SDB) at 37°C.

### Wheat Cultivars Selection Criteria

Six wheat varieties containing different combinations of gliadin coding alleles were selected from various parts of Pakistan (Supplementary Table S1). These varieties had different genomic characteristics and physiochemical properties.

### Assessment of Phytic Acid Degradation Potential

#### Qualitative Assay

For the qualitative degradation involving phytate, MRS agar medium was supplemented with sodium phytate, which was dissolved in sterilized distilled water and microfiltered using a 0.25 μm filter. A 3 μL suspension consisting of 10^7^–10^8^ CFU/ml was prepared for inoculation in the wells. After 24 h of incubation, the microbial colonies were washed using autoclaved water and petri plates were flooded with 2% (w/v) CoCl_2_ solution (Bae et al., 1999) and incubated for 5 min at 30°C. Thereafter, a solution consisting of equal volumes of ammonium molybdate solution [6.25% (w/v)] and ammonium metavanadate solution [0.42% (w/v)] replaced the CoCl_2_ solution on the plates. The plates were examined for the phytate hydrolysis zone after 10 min of incubation after removing the solution (Haros et al., 2007).

#### Quantitative Assay

Microbial isolates with vivid zone of degradation were analyzed for their efficiency to degrade phytic acid by spectrophotometric assessment at 530 nm (Helios Alpha Spectrophotometer, Thermo Scientific, United States). For assessment of phytase activity, a modified method of Naito (1975) was used to measure phytase-mediated release of inorganic orthophosphate from phytic acid. A reaction mixture was prepared containing 150 μL cell suspension and 600 μL substrate (3 mM sodium phytate dissolved in 0.2 M sodium acetate, pH 4.0), and incubated at 37°C (Shimizu, 1992). This reaction was stopped by the addition of 750 μL 5% C_2_HCl_3_O_2_. The inorganic orthophosphate was determined by adding 750 μL of color reagent, which was prepared freshly by mixing four volumes of ammonium molybdate [1.5% (w/v)] in a 5.5% (v/v) H_2_SO_4_ solution and one volume solution of a FeSO_4_ [2.7% (w/v)] (Sigma, F-7002).

### Proximate, Rheological and Metal Analysis of the Wheat Varieties

The six wheat varities were analyzed for water absorption, dough developing time, dough stability, dough tolerance or resistance and tolerance index according to AOAC (2000).

### Metal Analysis of Wheat Varieties by Proton Induced X-Ray Emission (PIXE)

Dried flour was taken for pellet formation and the pellet was placed in the PIXE apparatus. The concentration of various metals in flour was analyzed from the PIXE spectra by GUPIXWIN software package (Version SRIM-2008.04, University of Guelph, Canada). This provides a non-linear least square spectrum fitting, along with conversion of the fitted X-ray peak intensities into concentrations of elements, and a fundamental parameter method was used for quantitative analysis according to method of Mubark Ebrahim et al. (2014).

### Sourdough Fermentation

Six wheat varieties were subjected to fermentation with *S. cerevisiae*, *W. anomalus* QAUWA03, *E. mundtii* QAUSD01, consortium of *E. mundtii* QAUSD01 and *W. anomalus* QAUWA03. Commercially available baker’s yeast was used in 1.5% (w/v) concentration for wheat fermentation. Samples without fermentation were used as controls. Microbial cells were cultivated till the late exponential growth phase, for their usage toward sourdough fermentation. Fermentation of six wheat varieties flour was done according to the method of De Angelis et al. (2006a) with minor modifications. Briefly, thirty grams of wheat flour from each variety was mixed thoroughly with 36 mL sterilized double distilled water and a 14 mL suspension containing 5 × 10^8^ CFU/mL of one of the microbial strains to obtain 80 g of dough. Batters were incubated at 37°C for 48 h with stirring (200 rpm) following which the sourdough samples were immediately freeze-dried (Labconco freeze drier, United States) for further analysis.

### Determination of Phytic Acid by GC-MS

#### Sample Preparation

Twenty-five milligrams of freeze dried sample from sourdough was shaken with 200 μL of 12 M HCl for 4 h and then diluted with deionized water until pH 4 was reached and thereafter filtered through 0.45 μm membrane filter. Twenty microliters of sample were used for derivatization.

#### Derivatization

Derivatization of scyllo-inositol and phytic acid was done according to the method of March et al. (2001). Concentrations were estimated based on the calibration curve using silylated compounds of scyllo- and myo-inositol standards as the reference peaks.

### Sample Analysis

Phytic acid or salts of phytate are component of cereals located particularly in the bran portion of cereals. Gas chromatography-(GC)-mass spectrometry (MS) analysis for the detection of phytate degradation was used, which was based on the silylated reaction of hexamethyldisilazane and chlorotrimethylsilane with myo-inositol and scyllo-inositol used as internal standards. Under these parameters, the synthesis of hexamethylsilylinositol readily occurs. The method for phytic acid determination involves separation of phytic acid from free myo-inositol and scyllo-inositol followed by hydrolysis of phytic acid to myo-inositol. No peaks are observed if inositols are fully degraded by the fermentation. GC-MS analysis of phytic acid degradation was undertaken using an Agilent 7890A gas chromatograph coupled to a 5975C mass spectrometer and a DB-5MS column (J&W Scientific, United States). Helium gas was used as carrier gas with a flow rate of 1 mL/min. An injection volume of 1 μL was used with a split ratio of 10:1. The oven temperature was set at 72°C for 42 min and gradually increased to 315°C at a rate of 5°C/min and maintained for 12 min at this temperature. The injector inlet temperature was 295°C and the transfer line temperature was 285°C (March et al., 2001).

### Extraction and Preparation of Gliadin From Fermented Wheat Dough Samples

Gliadin was extracted from the lyophilized fermented wheat flour samples. Salt soluble proteins from fermented wheat samples were first removed by the extraction of 10 g freeze dried sample with 30 mL of 1 M sodium chloride in a shaker for 1 h. The samples were then centrifuged for 20 min at 4000 ×*g* and the resulting pellet was centrifuged at 3500 ×*g* for 20 min after washing with 40 mL of double distilled water. The resulting pellet was suspended in 30 mL of 70% ethanol and the mixture was incubated for 60 min at 60°C in shaking water bath. The gliadin in the supernatant fraction was lyophilized for further analysis.

### Fourier Transform Infrared Spectroscopy (FT-IR) Analysis of Gliadin Degradation

To determine the degradation of gliadin as assessed by FT-IR, 2 mg of lyophilized sample was mixed with 198.0 mg KBr. The mixture was ground in a mortar to be homogeneous and pressed into a thin slice. FT-IR spectra were recorded on an FT-IR spectrophotometer (Vector 33, Bruker Co., Germany) using an FT-IR cell, and the internal reflection element was a Zn–Se plate. Spectra were recorded as the average of 128 scans at 2 cm^-1^ resolution. The cell compartment was flushed with dry nitrogen during measurements. FT-IR Spectra were Fourier self-deconvoluted by Peak Fit v4.12 software. At first, small band in amide I and amide II region from 1500–1600 cm^-1^ to 1600–1700 cm^-1^ was chosen as this band could cause a misestimation of the peak maximum and intensity of the amide I band and amide II of gliadin in sourdoughs. A linear baseline between 1500 and 1700 cm^-1^ was formed, and the baseline was linearly corrected.

### Reverse Phase (RP)-High Performance (HPLC) Analysis of Gliadin Degradation

To determine the degradation of gliadin as assessed by RP-HPLC, hydrolyzed gliadin isolated from each sample was mixed with 0.1% trifluoroacetic acid (TFA) and centrifuged at 5000 ×*g* for 10 min. The resulting supernatant was filtered using 0.45 μm Millipore^TM^ membrane filters and stored at -18°C. The supernatant underwent RP-HPLC analysis according to the method of Antila et al. (1991). Analyses were carried out with HPLC (Model 126, Beckman, Brea, CA, United States) coupled with programmable solvent module for high-pressure delivery of solvent. Chromatographic spectral data was analyzed by the Gold System (ver. V810). For chromatographic separation, 100 mL of sample was injected into a protein and peptide reverse phase C18 column (250 × 4.6 mm, J.T. Baker Inc., United States) and run at room temperature. The sample was eluted at a flow rate of 0.8 mL/min with two buffer gradient system: solvent A, 0.1% TFA in water (v/v); solvent B, 0.11% TFA (60% acetonitrile/40% acidified water), which was increased linearly from 0 to 90% over 50 min. The elution was monitored at 215 nm.

### Pepsin Trypsin Digestion

The gliadin extract (120 mg) was dissolved in 20 mL of sodium acetate buffer (50 mM, pH 4). Pepsin (3200 U/mg; P-6887; Sigma-Aldrich, United States) and mixed with sample prior to incubation at 37°C under agitation for 2 h. Following the incubation, 142 mg of sodium phosphate was added, and the pH was adjusted to 7.0 using NaOH. Afterward, trypsin (2500 U/mg; T-7418; Sigma-Aldrich, St. Louis, MO, United States) was added and the solution was mixed and incubated for 2 h under agitation at 37°C. The resulting solution was heated at a temperature greater than 95°C for 10 min to stop the enzymatic reaction and the solution was lyophilized and stored at -20°C for further analyses.

### Caco-2 Monolayer Cell Culture

#### Intestinal Epithelial Cell Culture

The effects of the fermentation-mediated gliadin degradation were assessed on the viability of the monolayers of the Caco-2 intestinal epithelial cell line (American Cell Type Collection, HTB-37, Rockville, MD, United States; passage 23–40). Caco-2 cells were cultured in minimum essential medium (MEM; Gibco Invitrogen, Paisley, United Kingdom) supplemented with 10% fetal bovine serum (FBS; Gibco Invitrogen), 1% non-essential amino acids (NEAA; Gibco Invitrogen), 0.1% penicillin–streptomycin (Gibco Invitrogen), sodium bicarbonate (Gibco Invitrogen) and sodium pyruvate (Sigma-Aldrich, Seelze, Germany). Cells were cultured at 37°C in 5% CO_2_ and passaged after 5–7 days, when they were reached 80% confluency.

### Direct Immunofluorescence and Zonula Occludens 1 (ZO1) Localization Migration in Intestinal Cell Monolayers

After Caco-2 cells were reached 80% confluency at 25th passage, they were grown on 8-chamber slides and incubated with PT-gliadin from the six wheat varieties fermented with *E. mundtii* QAUSD01 and *W. anomalus* QAUWA03 consortium to assess effects on ZO1 localization. The monolayers were fully developed after 4 days incubation in media. The gliadin fractions were added at a concentration of 1 mg/mL to the Caco-2 cell monolayers. Caco-2 cells were then gently washed with phosphate buffer saline (PBS) and permeation was done with methanol for 2 min at -20°C. The Caco-2 monolayers were then washed with PBS three times and incubated with primary antibodies (FITC-conjugated anti-ZO1 monoclonal antibody; Zymed Laboratories Inc., San Francisco, CA, United States). The slides were washed twice with PBS after 60 min of incubation, air-dried and observed under a fluorescent microscope (Olympus fluorescent microscope, BX60 Olympus Corporation, Tokyo, Japan).

### Immunofluorescence Microscopy

To evaluate the impact of fermented gliadin on tight junction proteins, Caco-2 cells were grown onto eight chamber glass slides (BD Biosciences, Erembodegem, Belgium) for immunofluorescence. After 4 days of culture, Caco-2 monolayers were washed twice with Hank’s balanced salt solution (HBSS; Gibco Invitrogen) and incubated overnight in MEM media supplemented with 10% FBS, 1% NEAA, sodium pyruvate, sodium bicarbonate. Afterward Caco-2 cells were washed twice with PBS and 4% paraformaldehyde (Merck, Darmstadt, Germany) used for fixation. A solution of 0.1% Triton X-100 (Sigma-Aldrich, St. Louis, MO, United States) was used to permeate the cells. For the visualization of membrane ruffle formation, cells were stained for intracellular F-actin with phalloidin–fluorescein isothiocyanate (Sigma-Aldrich, St. Louis, MO, United States) and observed under a fluorescent microscope (Olympus fluorescent microscope, BX60 Olympus Corporation, Tokyo, Japan).

### *Trans*-Epithelial Resistance (TER)

Caco-2 cells were grown on Millicell Culture inserts (Millipore Corporate, Billerica, MA, United States) until confluency for 21 days. The Millicell-ERS volt-ohm meter (Millipore Corporate, Billerica, MA, United States) was used for the measurement of resistance in the cell monolayers. When the TER value exceeded 600 ohms/cm^2^, cells were considered confluent. The monolayers were double washed and incubated for 24 h in MEM media. After addition of PT-BSA and PT-gliadin, TER was measured at the 1, 2, 4, 12, and 24 h time points after refreshing the media. Sample analysis was carried out in duplicate and three independent experiments were carried out.

### Statistical Analysis

SPSS version 13.0 for Windows (SPSS Inc., United States) was used for the analysis of experimental results. One-way ANOVA was performed on the phytic acid degradation results. Two-way ANOVA was applied for the TEER values. Tukey’s test was used to discriminate between the mean differences. Differences between data were considered significant at *P* < 0.05.

## Results

### *In vitro* Phytic Acid Degradation

**FIGURE 1:**
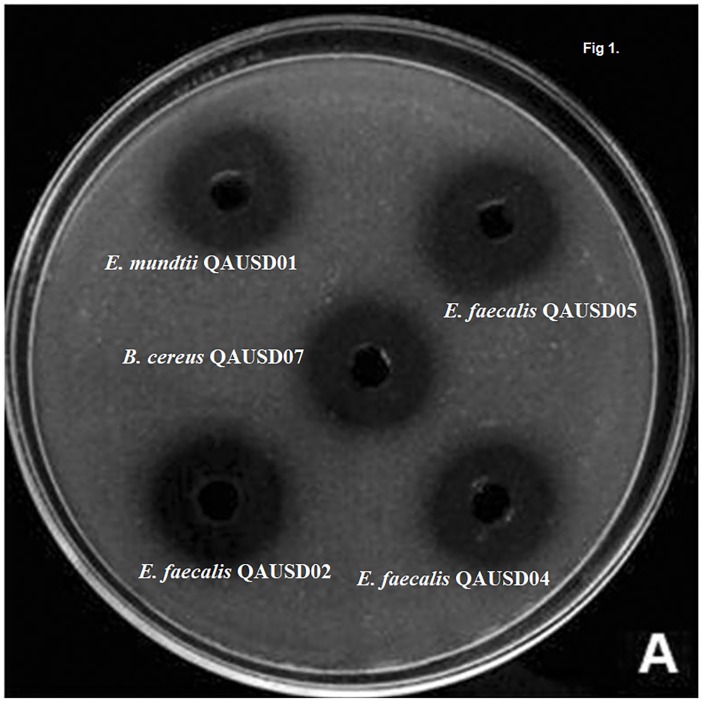
Clearance zones indicates the phytic acid degradation and high clearance zones indicates more phytic acid degradation. These strains were isolated in previous experiment and these strains exhibited significant gliadin degradation.

The 19 isolates were analyzed for phytic acid degradation by the plate assay. The *E. mundtii* QAUSD01*, E. faecalis* QAUSD02, *E. faecalis* QAUSD04*, E. faecalis* QAUSD05*, E. faecalis* QAUSD06*, Bacillus cereus* QAUSD07 strains showed zones of phytic acid hydrolysis while *E. megaterium* QAUSD03 demonstrated no zones of degradation in the second screening and the remaining isolates had no zones of degradation in the first screening. *E. faecalis* QAUSD05 (LAB 12) showed the maximum zone of hydrolysis, which was followed by *E. mundtii* QAUSD01 (LAB 13) and *B. cereus* QAUSD07 (LAB 18), *E. faecalis* QAUSD02 (LAB 6) and *E. faecalis* QAUSD04 (LAB 8) (Figure 1).

**FIGURE 2:**
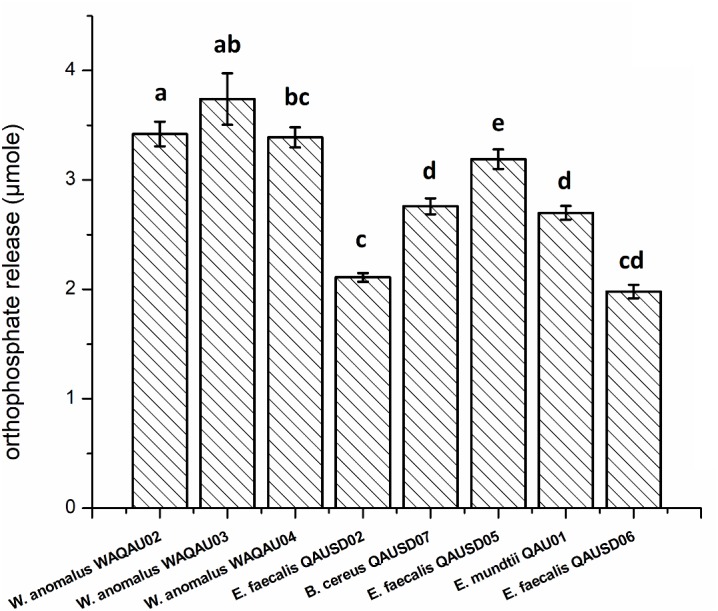
Orthophosphate release in the various gliadin-degrading strains Bars not sharing the same letters are significantly different from each other by the Tukey’s test.

For quantitative measurement of phytic acid hydrolysis, the six isolates along with three yeast strains were analyzed by spectrophotometry using sodium phytate as substrate (Figure 2). *E. faecalis QAUSD05* showed the maximum ability to degrade sodium phytate among all six strains by releasing 3.19 μM of phosphorus while *E. faecalis QAUSD02*, *E. faecalis QAUSD06*, *Bacillus cereus* QAUSD07 and *E. mundtii* QAUSD01 released 2.11, 1.98, 2.70, and 2.76 μM of phosphorus, respectively. *E. faecalis* QAUSD04 (LAB 8) had minimal hydrolysis capability. The yeast strains *W. anomalus* QAUWA02, *W. anomalus* QAUWA03 and *W. anomalus* QAUWA04 demonstrated a similar capacity to degrade sodium phytate as they released 3.42, 3.74, and 3.39 μM phosphorus, respectively.

### Physiochemistry and Rheological Analysis

Proximate analysis and the rheological results of the wheat cultivars are shown in Supplementary Table S2. Proximate analysis of the wheat varieties showed that *Barani 83* had the maximum protein, fat and ash content whereas *Shafaq 2006* showed the lowest values of protein and ash. *Barani 83* had the maximum dough development time (9 min) and dough stability (12.5 min) while *Seher 2006* had the shortest dough development time (4.5 min) and smallest percent of water absorption. The maximum water absorption was observed in *Chakwal 97* whereas the lowest values were seen in *Seher 2006.* Mineral analysis of the wheat varieties was performed by proton-induced X-ray emission (Supplementary Table S3). *Barani 83* showed the highest concentration of Mg and Fe while *Lasani 08* had the lowest concentration of Mg and *Bars 2009* demonstrated the lowest concentration of Fe.

### Gliadin Degradation by Wheat Dough Fermentation

**FIGURE 3:**
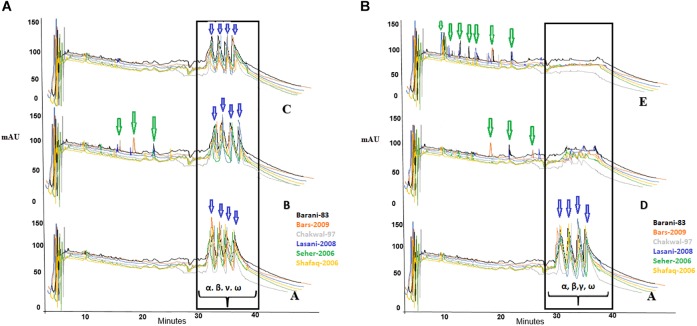
**(A)** A denotes control sample and the peaks shows no degradation of gliadin fractions; B denotes the fermentation of wheat flours with *W. anomalus* QAUWA03; C denotes the fermentation of wheat flours with *S. cerevisiae* fermentation. Different colors express the various wheat varieties. Arrows indicates formation of peptides and amino acids. No substantial degradation was observed in these samples. In this graph α, β, γ, and ω denotes the types of gliadin in wheat varieties. **(B)** A denotes control sample and the peaks shows no degradation of gliadin fractions; D denotes the fermentation of wheat flours with *E. mundtii* QAUSD01; E denotes the fermentation of wheat flours with *W. anomalus* QAUWA03 and *E. mundtii* QAUSD01 consortia. Maximum gliadin degradation was observed in consortia fermented wheat flours. Arrows indicates formation of peptides (<33 mer) and amino acids. Different colors express the various wheat varieties. In this graph α, β, γ, and ω denotes the types of gliadin in wheat varieties.

The effect of fermentation with various microbial isolates and consortia on gliadin degradation analyzed by RP-HPLC is shown in Figure 3. In the control samples, gliadin eluted between 30 to 40 min with no notable differences seen among the wheat cultivars. Degradation percentage of different wheat cultivars after 48 h fermentation is presented in Table 1. Fermentation with commercially available *S. cerevisiae* showed no evidence of gliadin degradation of any of six wheat cultivars. This latter finding could be due to omission of sugar to facilitate the growth of this commercial yeast, which is normally added during industrial fermentation or for the production of bread. The *W. anomalus* QAUWA03 fermentation showed minimal effects on the degradation of the gliadin fractions with the wheat variety *Chakwal 97* showing the greatest degradation. Similarly, low levels of gliadin degradation were noted with *E. mundtii* QAUSD01 with the maximum degradation of gliadin noted in *Barani 83*. In contrast, fermentation of the wheat varieties by the consortia of *E. mundtii* QAUSD01 and *W. anomalus* QAUWA03 showed significant gliadin degradation indicating synergistic effects on gliadin hydrolysis. Consortia fermentation caused maximum degradation in *Shafaq 2006* with relatively lower degradation was observed in *Barani 83*. The other varieties of *Bars 2009*, *Chakwal 97*, *Lasani 2008*, and *Seher 2006* had similar levels of gliadin degradation. Consortia fermentation also resulted in generation of small peptides and amino acids following degradation of gliadin.

**Table 1:** Percentage degradation of gliadin in 30 samples after 48 h fermentation.

Wheat cultivars	Control (A)	*S. cerevisiae* fermentation (B)	*W. anomalus* QAUWA03(C)	*E. mundtii* QAUSD01 (D)	*W. anomalus* QAUWA03 and *E. mundtii* QAUSD01 consortia(E)
*Barani 83*	7 ± 1.7	11 ± 2.1	17 ± 1.6	28 ± 2.4	89 ± 4.6
*Bars 2009*	6 ± 1.4	12 ± 1.9	19 ± 1.7	27 ± 2.6	91 ± 4.3
*Chakwal 97*	7 ± 1.5	10 ± 2.3	20 ± 1.8	26 ± 2.2	91 ± 3.8
*Lasani 2008*	7 ± 1.8	11 ± 2.1	19 ± 1.9	24 ± 2.3	90 ± 5.2
*Seher 2006*	8 ± 1.4	13 ± 2.4	18 ± 2.1	23 ± 2.5	92 ± 4.9
*Shafaq 2006*	6 ± 1.7	10 ± 1.9	16 ± 1.4	26 ± 2.1	93 ± 5.7


*Results are presented as means ± SD (*n* = 3)*.

### Gliadin Degradation Assessment by FT-IR Spectroscopic Analysis

**FIGURE 4:**
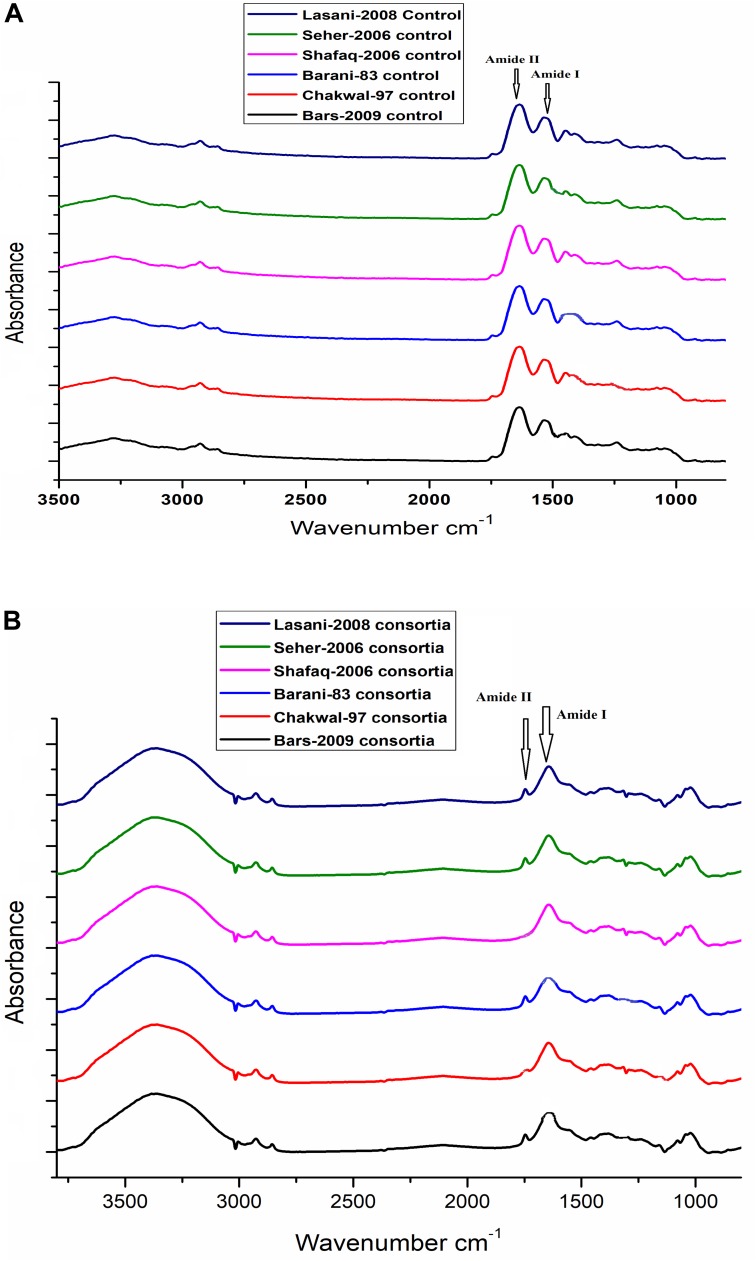
**(A)** No significant difference was observed among all wheat cultivars (control). Significant peaks of gliadin were observed in amide I and amide II region. **(B)** No significant peaks were observed in amide I and amide II region. However, no significant difference was observed among all wheat cultivars fermented with *E. mundtii* QAUSD01 and *W. anomalus* QAUWA03 consortium.

In sourdough fermentation, degradation of protein is among the key factors influencing the overall sourdough food quality. Protein hydrolysis in cereal fermentation has been investigated extensively to improve flavor formation in baking and as an approach to decrease protein and peptide to concentrations considered to be safe for celiac patients. The change in structure of gliadin proteins in wheat sourdough fermentation for 48 h was determined using Fourier transform infrared spectroscopy, and then the resultant spectra showed Fourier self-deconvolution of the amide I and amide II bands in the regions of 1600–1700 cm^-1^ and 1500–1600 cm^-1^, respectively. Significantly different spectra in the amide I and amide II bands for gliadin from sourdough fermented with *W. anomalus* QAUWA03 and *E. mundtii* QAUSD01 consortium was noted (Figure 4B) in comparison to the control dough (Figure 4A). No significant degradation was observed in dough fermented with *S. cerevisiae* with *E. mundtii*, and sourdough fermented with *W. anomalus* QAUWA03 after 48 h of fermentation (data not shown). The loss of secondary structure in *W. anomalus* QAUWA03 and *E. mundtii* QAUSD01 consortium samples during fermentation indicates that the flexibility of gliadin in sourdough increased, which could facilitate gliadin degradation during fermentation. The modified secondary structure of gliadin reveals proteolysis of gliadin proteins as no peaks were observed in amide I and amide II region, which is a clear indication of gliadin degradation in consortium fermented sourdough samples. No significant difference in gliadin degradation was demonstrated among all six fermented wheat cultivars.

### Phytic Acid Degradation (*in situ*) in Fermented Dough

**FIGURE 5:**
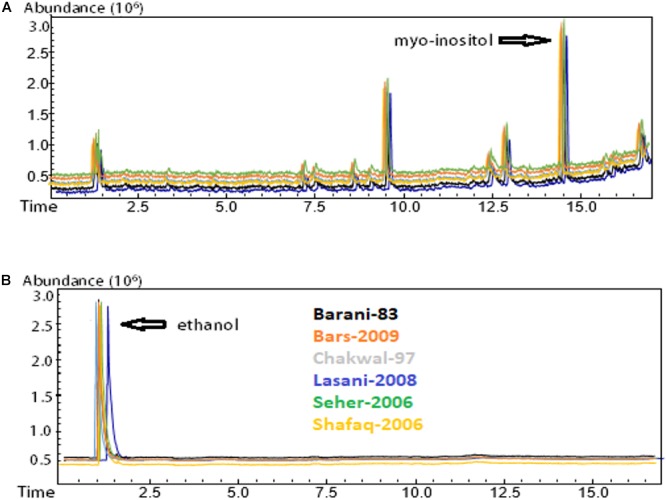
Phytic acid degradation determination by GC-MS analysis. **(A)** Control sample without fermentation; **(B)** Consortia fermented samples. Myo-inositol was completely degraded in all consortia fermented wheat verities. Different colors express the various wheat varieties.

The 12 samples showing significant gliadin degradation were selected for analysis of phytic acid degradation. GC-MS analysis of phytic acid degradation showed that phytic acid was completely degraded by consortia fermentation while no degradation of phytic acid was observed in the control samples (Figure 5). All six wheat varieties demonstrated a complete lack of phytic acid when fermented by the *E. mundtii* QAUSD01 and *W. anomalus* QAUWA03 consortium. It is likely that the gliadin degradation was not only attributable to phytases produced by the consortia as low pH and the activation of phytases naturally present in flour could have been involved to degrade phytic acid in the presence of water soaking, particularly since phytic acid is not stable at very low pH (Helbig et al., 2003).

### Direct Immunofluorescence and ZO1 Migration in Intestinal Cell Monolayers

**FIGURE 6:**
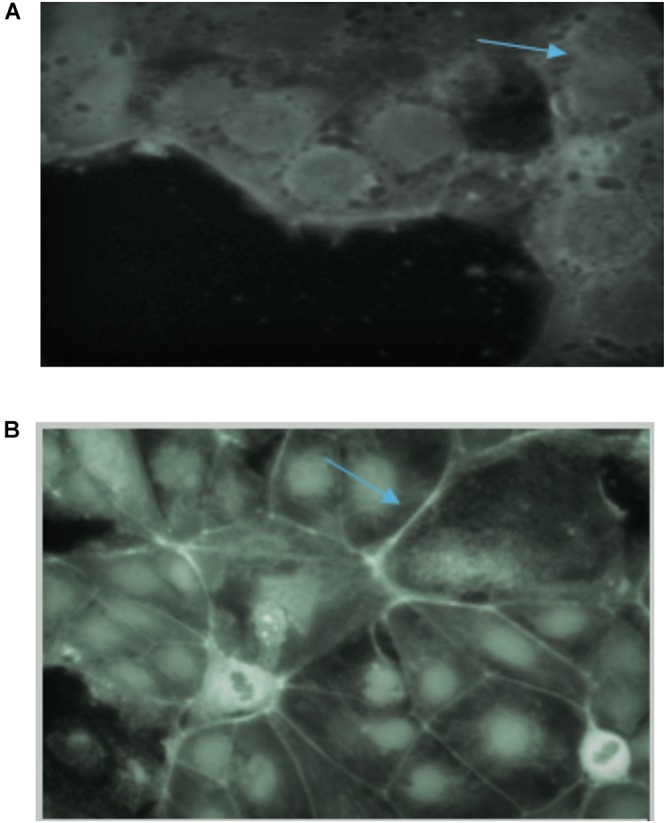
**(A)** Effect of gliadin on tight junctions of Caco-2 monolayers in control sample (no fermentation). Arrow indicates the destruction of tight junctions of monolayers due to gliadin toxicity. **(B)** Effect of consortia fermented *Lasani 2008* wheat cultivar on tight junctions of Caco-2 monolayers. Arrow indicates no perturbation on the tight junctions.

The tight junction protein ZO-1 was analyzed to establish whether the fermentation of consortia exerted any adverse effect on tight junctions. Tight junctions in the control samples showed a curvy appearance whereas the tight junctions were shown to be destroyed following treatment with all the wheat cultivars, which is likely due to gliadin toxicity (Figure 6A). In terms of the consortia fermentation for the six wheat varieties, only fermentation using *Lasani 2008* showed no detrimental impact on tight junction proteins (Figure 6B), which indicates that the toxic gliadin residues were completely degraded following this fermentation. In contrast, the other fermented wheat varieties demonstrated a negative impact on the tight junctions of the monolayers as those consortia-fermented samples showed straightened tight junctions as opposed to the smooth appearance in the controls. Wheat fermentation with *E. mundtii* QAUSD01, *W. anomalus* QAUWA03 and the commercial *S. cerevisiae* strain showed no significant effects on tight junctions, smoothness and appearance (data not shown).

### Effect of Fermentation on Gliadin-Induced Membrane Ruffle Formation

**FIGURE 7:**
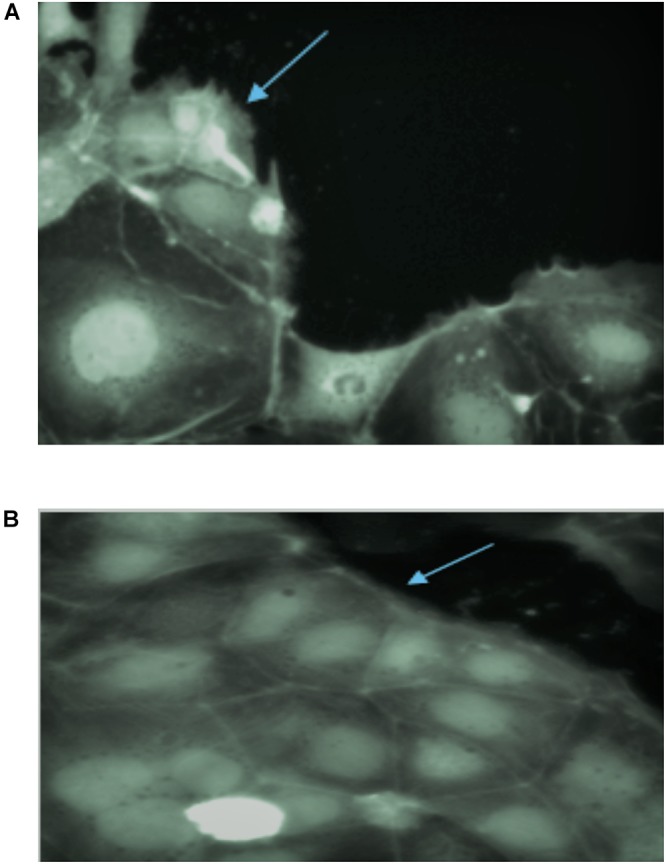
**(A)** Ruffle formation by gliadin in control samples on Caco-2 monolayers. Arrow indicates the ruffle formation on monolayers due to toxic effect of gliadin. **(B)** No ruffle formation was observed in consortia fermented *Lasani 2008* gliadin on Caco-2 monolayers. Arrow indicates smooth monolayer without any ruffle formation.

Gliadin has been reported to induce distinct membrane ruffling on the edges of Caco-2 cell monolayers clusters (Lindfors et al., 2008). PT-gliadin induced significant membrane ruffle formation in Caco-2 cells (Figure 7A) as membrane ruffles covered most of the area of monolayers. PT-gliadin of all wheat varieties treatment increased the ratio of cell cluster edge covered by ruffles. This type of ruffle formation was also observed in all wheat varieties fermented with *S. cerevisiae*, *E. mundtii* QAUSD01 and *W. anomalus* QAUWA03 alone (data not shown). Two wheat varieties (*Lasani 2008* and *Seher 2006*) fermented with consortia of *E. mundtii* QAUSD01 and *W. anomalus* QAUWA03 showed reduced ruffle formation (Figure 7B). These latter results indicate that the toxic gliadin fragments appeared to be completely degraded following digestion in these two wheat varieties as opposed to the other varieties.

### Fermentation Counteracts the Gliadin-Induced Increase in Epithelial Cell Permeability

**FIGURE 8:**
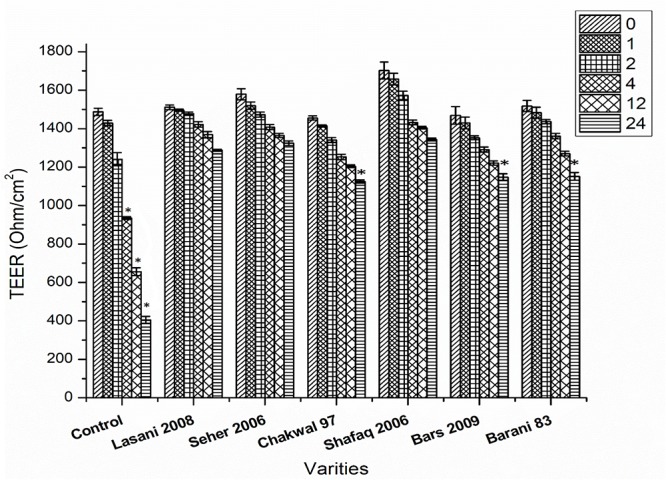
Effect of consortia fermentation on TER value. Asterisks indicate significant different in TER values of wheat cultivars after 24 h by analysis of variance (ANOVA) and significance was determined by Tukey’s test (*p* < 0.05).

**FIGURE 9:**
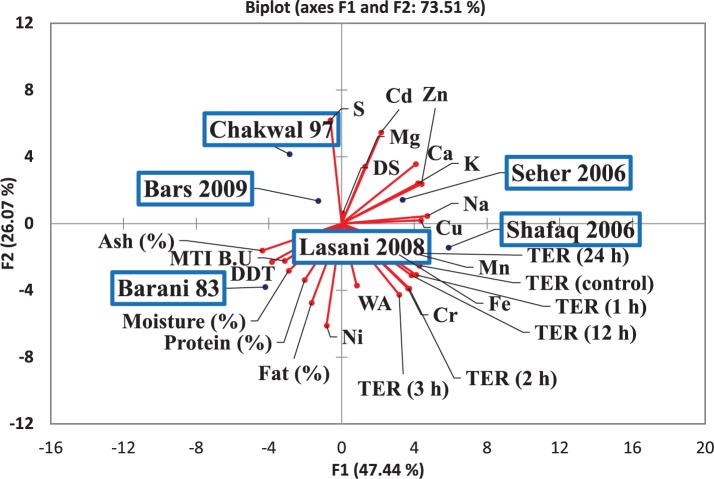
Score and loading plot of the principal component analysis (PCA) carried out on the covariance matrix of wheat varieties, rheological properties, metals analysis and TER values.

The efficacy of consortia fermentation of different wheat varieties to hinder the gliadin-induced increase in Caco-2 cell permeability as assessed by TER is shown in Figure 8. A significant change in TER values was observed in the control samples after 4, 12, and 24 h. All wheat varieties fermented with consortia showed a change in TER values after 24 h as compared to control values with non-significant differences in TER observed among the varieties at all time points. This latter finding indicates that consortia fermentation inhibited the gliadin-induced decrease in TER, which signifies minimal adverse effects were exerted on the monolayer structures. In contrast, single strain fermentation did not inhibit the decrease in the gliadin induced TER (data not shown). Principle component analysis (PCA) was conducted to evaluate the correlation among the wheat varieties, rheological properties, metals analysis and TER values (Figure 9). The Scores analysis showed that the two first principal components explained 73.51% of the total variance, which the PC1 and PC2 were 47.44 and 26.07%, respectively. All wheat varieties were clearly differentiated from each other in PCA plot. TER values have positive correlation with *Shafaq 2006.* While negative correlation was observed in *Chakwal 97.*

## Discussion

The present study was carried out with the aim to isolate bacterial and fungal strains that can concurrently degrade phytic acid and gliadin among a variety of wheat varieties. Significantly, the results showed for the first time that fermentation carried out by consortia of *E. mundtii* QAUSD01 and *W. anomalus* QAUWA03 could induce both phytic acid and gliadin degradation. Earlier studies have noted that certain probiotic strains can degrade phytic acid (Reale et al., 2007; García-Mantrana et al., 2016) and that introduction of different probiotic strains in sourdough fermentation results in a varying efficacy of gliadin hydrolysis (De Angelis et al., 2006b; Gobbetti et al., 2007) but concurrent degradation of phytic acid and gliadin has not been previously shown. Enzyme preparations (Rollan et al., 2005), cell extracts (Di Cagno et al., 2002) and intact probiotic preparations (De Angelis et al., 2006b) have also been shown to degrade gliadin peptides indicated to play a pathogenic role in CD. In that regard, no adverse effects on the Caco-2 cell monolayers was shown from the gliadin peptide fractions derived from wheat varieties fermented with consortia of *E. mundtii* QAUSD01 and *W. anomalus* QAUWA03, which demonstrates the potential of this fermentation to protect against CD risk. This consortium could thus be a potential candidate for industrially fermented bread. In that regard, *W. anomalus* could be particularly useful as it has also been shown to induce a greater rate of carbon dioxide production as compare to standard *S. cerevisiae* strains (Walker, 2011).

The physiochemical and rheological properties of wheat are largely attributed to gliadin and glutenin, which constitute 80% of total wheat proteins (Tatham and Shewry, 1995). It has been suggested that the gliadins generally contribute to dough viscosity and glutenins contribute to dough elasticity (Khatkar and Schofield, 1997). These two proteins are also found in various ratios, which imparts different rheological properties to wheat varieties. In the present work, gliadin was degraded same extent in all wheat varieties. This latter result could be due to the high ratio of gliadin in those varieties as those varieties also revealed high viscosity compared to elasticity, which is the indication of high gliadin content as well as relatively high concentrations of prolamins (Uthayakumaran et al., 1999).

In summary, the data presented demonstrates that the *E. mundtii* QAUSD01 and *W. anomalus* QAUWA03 consortia have the potential to degrade gliadin in selected wheat varieties. The above fermentation was also shown to alleviate gliadin-induced insult to Caco-2 cell monolayers mediated by the *Lasani 2008* and *Seher 2006* wheat varieties, which coincides with previous work showing protective effects of certain probiotic microbial strains against some gluten- and gliadin-mediated deleterious effects (Eggert et al., 2011). Thus, probiotic microbes in sourdough fermentation have the potential for industrial use toward degradation of toxic gliadin fractions during food processing. The breakdown of phytic acid by *E. mundtii* QAUSD01 and *W. anomalus* QAUWA03*-*mediated fermentation could lead to additional health promoting benefits by improvement in the bioavailability of essential trace minerals. These strains could also be investigated in future studies for their potential as oral supplements to alleviate CD as well as enhancing iron absorption from fermented wheat products and use in the baking industry for production of sourdough bread. Certain research challenges are still needed to be addressed toward industrial applications of the identified probiotic strains in fermented breads and cereal foods. Such considerations include optimization of the manufacturing conditions to evaluate the storage stability of the probiotic strains, the growth capacity and productivity of the sourdough starter culture as well as organoleptic characteristics of the final product.

## Author Contributions

MI and SK conceived and designed the experiments. HS and BAz performed the experiments. UQ, BAl, RF, SG, and HS analyzed the data. MI and SK drafted the manuscript. All authors read and approved the final manuscript.

## Conflict of Interest Statement

The authors declare that the research was conducted in the absence of any commercial or financial relationships that could be construed as a potential conflict of interest.
