# Supplementary material for: Wheat Fermentation With Enterococcus mundtii QAUSD01 and Wickerhamomyces anomalus QAUWA03 Consortia Induces Concurrent Gliadin and Phytic Acid Degradation and Inhibits Gliadin Toxicity in Caco-2 Monolayers
Source: Front Microbiol. 2019 Feb 12;9:3312. doi: 10.3389/fmicb.2018.03312 (PMC6396733; doi:10.3389/fmicb.2018.03312)
Supplement: Supplementary file 1 [file Data_Sheet_1.docx]

**Supplementary Material**

**Supplementary Table S1**.

Wheat Varieties with genetic and agronomic information

| **Variety Name** | **Release Year** | **Parentage** | **Pedigree** | **Yield Potential (Kg/hectare)** | **Ecology** | **Glia-A1** | **Glia-A1** | **Glia-A1** |
| --- | --- | --- | --- | --- | --- | --- | --- | --- |
| *Bars-2009* | 2009 | PFAU/SERI//BOW | CMSS97M00306S-0P5M-095Y-90M 010Y | 5613 | Rainfed areas | a | a | A |
| *Lasani-2008* | 2008 | LUAN/KOH-97 | PBP.29645-14A-18A-8A-4A-2A-0A | 6200 | Irrigated areas | b | a | a |
| *Seher-2006* | 2006 | CHILL/2* STAR/4/BOW//BUC/PVN/3/2*VEE#10 | CM SS95Y00645-100Y-200M-17Y-10M-0Y-0PAK | 7000 | Irrigated areas | b | a | b |
| *Chakwal-97* | 1998 | BUC'S'/FCT'S' | CM64663-7M-0Y-0M-7Y-0M | 5500 | Rainfed areas | a | b | a |
| *Barani-83* | 1983 | BB/GLL/3/GTO/7C//BB/CN0 | CM32347-3M-1Y-1M-1Y-1K-0A-0PAK | 5200 | Rainfed areas | a | b | b |
| *Shafaq-2006* | 2006 | V 87094(LU 26/HD 21790/ 2*INQALAB 91 | PB 28633P-2A-6A-0A | 6000 | Irrigated areas | b | b | a |

**Supplementary Table S2**.

Proximate and rheological analysis of wheat varieties

| **Wheat Variety** | **Moisture (%)** | **Fat (%)** | **Ash(%)** | **Protein (%)** | **DDT**  **(min)** | **WA (%)** | **MTI (B.U*)** | **DS**  **(min)** |
| --- | --- | --- | --- | --- | --- | --- | --- | --- |
| ***Seher 2006*** | 10.2 | 2.2 | 1.40 | 13.5 | 4.5 | 55.5 | 38 | 06.1 |
| ***Shafaq 2006*** | 09.7 | 2.5 | 1.22 | 12.4 | 5.5 | 56.0 | 25 | 04.5 |
| ***Chakwal 97*** | 10.0 | 2.3 | 1.50 | 12.7 | 7.5 | 65.0 | 35 | 05.8 |
| ***Barani 83*** | 11.0 | 2.8 | 1.67 | 14.3 | 9.0 | 62.5 | 45 | 12.5 |
| ***Lasani 2008*** | 11.4 | 2.3 | 1.50 | 13.0 | 6.5 | 58.0 | 40 | 11.3 |
| ***Bars 2009*** | 11.0 | 2.4 | 1.40 | 12.5 | 5.5 | 63.0 | 30 | 09.8 |
| DDT; Dough Development Time, WA; Water Absorption, MTI; Mixing Tolerance Index, DS; Dough Stablity  *Brabender Units | | | | | | | | |

**Supplementary Table S3.**

Mineral Analysis of Wheat Varieties (micrograms per Kilogram)

| **Elements** | ***Barani 83*** | ***Chakwal 97*** | ***Shafaq 2006*** | ***Lasani 08*** | ***Seher 2006*** | ***Bars 2009*** |
| --- | --- | --- | --- | --- | --- | --- |
| **Ca** | 459 | 536 | 281 | 249 | 289 | 401 |
| **Mg** | 1627 | 1843 | 2058 | 1842 | 1980 | 2245 |
| **S** | 1208 | 1222 | 1216 | 1203 | 1225 | 1116 |
| **K** | 5012 | 5848 | 6214 | 5185 | 5950 | 5274 |
| **Na** | 27 | 26 | 23 | 16 | 20 | 21 |
| **Cr** | 10 | 12 | 8 | 9 | 10 | 8 |
| **Mn** | 52 | 56 | 51 | 52 | 55 | 49 |
| **Fe** | 57 | 49 | 44 | 58 | 51 | 47 |
| **Ni** | 25 | 25 | 13 | 39 | 37 | 23 |
| **Cu** | 6 | 8 | 9 | 6 | 7 | 8 |
| **Zn** | 42 | 36 | 29 | 16 | 28 | 34 |
